# The added value of SPECT/CT lymphoscintigraphy in the initial assessment of secondary extremity lymphedema patients

**DOI:** 10.1038/s41598-023-44471-2

**Published:** 2023-11-09

**Authors:** Hai-Jeon Yoon, Kyong-Je Woo, Ji-Young Kim, Seo Young Kang, Byung Seok Moon, Bom Sahn Kim

**Affiliations:** 1 Department of Nuclear Medicine, Ewha Womans University College of Medicine, Seoul, Korea; 2 Department of Plastic Surgery, Ewha Womans University College of Medicine, Seoul, Korea

**Keywords:** Oedema, Diagnostic markers

## Abstract

An added value of SPECT/CT over planar lymphoscintigraphy for initial staging in patients with secondary extremity lymphedema was investigated. Furthermore, we developed a hybrid SPECT/CT classification combining dermal backflow (DBF) of SPECT and honeycomb pattern (HP) of CT, correlated it with lymphoscintigraphic staging and clinical severity. Forty-one patients with secondary extremity lymphedema who underwent lymphoscintigraphy with SPECT/CT were included retrospectively. The severity of extremity lymphedema was assessed using CT volumetry. Lymphoscintigraphic findings were evaluated using the Taiwan Lymphoscintigraphy Staging (TLS), and CT-based and SPECT-based quantitative analysis were performed. TLS was performed by planar scintigraphy only and with SPECT/CT, respectively. The SPECT/CT findings were classified into DBF−/HP−, DBF+/HP−, DBF+/HP+, and DBF−/HP+. Based on these findings, patients were categorized into five classes: Class 1 = DBF−HP− entire limb, Class 2 = DBF+/HP− proximal/distal limb without DBF+/HP+ or DBF−/HP+, Class 3 = DBF+/HP+ proximal/distal limb without DBF−/HP+, Class 4 = Mixed DBF+/HP+ and DBF−/HP+ in proximal/distal limb, Class 5 = DBF−/HP+ entire limb. Adding SPECT/CT to planar scintigraphy showed a 15.4% modification rate in lymphoscintigraphic staging. HP volume ratio significantly increased as clinical severity and lymphoscintigraphic staging increased, while DBF volume ratio increased with severity and followed expected patterns according to lymphoscintigraphic staging. Hybrid SPECT/CT lymphoscintigraphic classification showed strong positive correlation with clinical severity and TLS. Our results demonstrated substantial modification of lymphoscintigraphic staging by adding SPECT/CT to a conventional planar scintigraphy. In addition, a hybrid SPECT/CT is expected to provide new indicators reflecting lymphoscintigraphic staging and clinical severity by providing both of functional DBF and anatomical HP information.

## Introduction

Lymphedema is a progressive pathology characterized by the accumulation of lymphatic fluid in the interstitial tissues, leading to inflammation, adipose tissue hypertrophy, and fibrosis^1^. It can occur as a primary or secondary disorder, the latter being the most common cause and often resulting from the treatment of malignancy^2^. Accurate diagnosis and staging of lymphedema are essential for appropriate management and prognosis, but it can be challenging due to the complex anatomy and physiology of the lymphatic system.

Lymphoscintigraphy is a widely used imaging technique for evaluating lymphatic function and identifying the extent and severity of lymphedema^3^. However, it has some limitations, such as poor spatial resolution and difficulty in distinguishing between lymphatic and non-lymphatic structures, which can affect the interpretation of results. Recently, hybrid single photon emission computed tomography/computed tomography (SPECT/CT) imaging has emerged as a valuable tool in nuclear medicine, offering the benefits of both modalities and enabling more accurate and precise diagnosis^4^.

In this study, we aimed to investigate the added value of SPECT/CT over planar lymphoscintigraphy in the initial staging of secondary extremity lymphedema. We also developed a novel classification system based on a combination of SPECT and CT findings, namely dermal backflow (DBF) and honeycomb pattern (HP), respectively, and correlated it with lymphoscintigraphic staging and clinical severity.

## Results

### Demographic data

**Figure 1:**
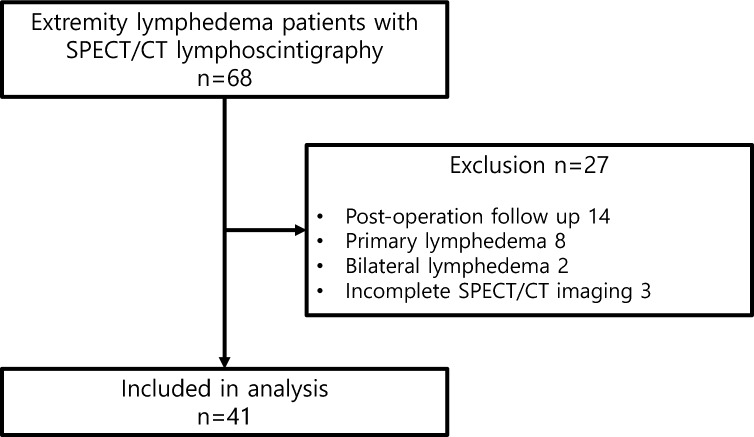
Flow chart of subject selection.

A total of 68 patients with extremity lymphedema underwent SPECT/CT lymphoscintigraphy in our center from April 2022 to December 2022. Of these, 14 patients who underwent imaging for follow-up after reconstructive surgery of the lymphatic system, eight patients with primary lymphedema, two patients with bilateral lymphedema, and three patients with incomplete imaging were excluded. Finally, a total of 41 unilateral, secondary extremity lymphedema patients (38 women, 3 men) who underwent SPECT/CT lymphoscintigraphy for initial assessment were included in the analysis (Fig. 1). Nineteen (46.3%) and 22 (53.7%) patients had lymphedema of the upper and lower extremities, respectively. The clinical characteristics of patients are summarized in Table 1.

**Table 1:** Clinical characteristics of patients.

	Total population (N = 41)	Upper extremity (N = 19)	Lower extremity (N = 22)	p-value
Age	60 (52, 63)	53 (45.5, 60)	61.5 (58, 66.75)	0.016
BMI	23.58 (21.08, 26.98)	23.31 (21.97, 25.39)	24.44 (20.45, 27.54)	0.574
Duration (months)	60.83 (24.33, 121.67)	36.5 (10.08, 109.5)	73 (25.85, 121.67)	0.180
Sex				
Female	38 (92.68%)	19 (100%)	19 (86.36%)	0.235
Male	3 (7.32%)	0 (0%)	3 (13.64%)	
ISL stage				
1	4 (9.76%)	3 (15.79%)	1 (4.55%)	0.321
2	36 (87.8%)	16 (84.21%)	20 (90.91%)	
3	1 (2.44%)	0 (0%)	1 (4.55%)	
History of compression				
Absence	17 (41.46%)	12 (63.16%)	5 (22.73%)	0.021
Presence	24 (58.54%)	7 (36.84%)	17 (77.27%)	
History of cellulitis*				
Absence	22 (55%)	13 (68.42%)	9 (42.86%)	0.192
Presence	18 (45%)	6 (31.58%)	12 (57.14%)	
Type of malignancy				
Breast cancer	19 (46.34%)	19 (100%)	0 (0%)	< 0.001
Cervical cancer	11 (26.83%)	0 (0%)	11 (50%)	
Endometrial cancer	4 (9.76%)	0 (0%)	4 (18.18%)	
Ovarian cancer	3 (7.32%)	0 (0%)	3 (13.64%)	
Other cancers	4 (9.76%)	0 (0%)	4 (18.18%)	
Radiotherapy				
Not done	12 (29.27%)	2 (10.53%)	10 (45.45%)	0.035
Done	29 (70.73%)	17 (89.47%)	12 (54.55%)	

This table was summarized as appropriate according to normality test.

*History of cellulitis is missed in one patient.

### Changes of lymphoscintigraphic staging by adding SPECT/CT

**Figure 2:**
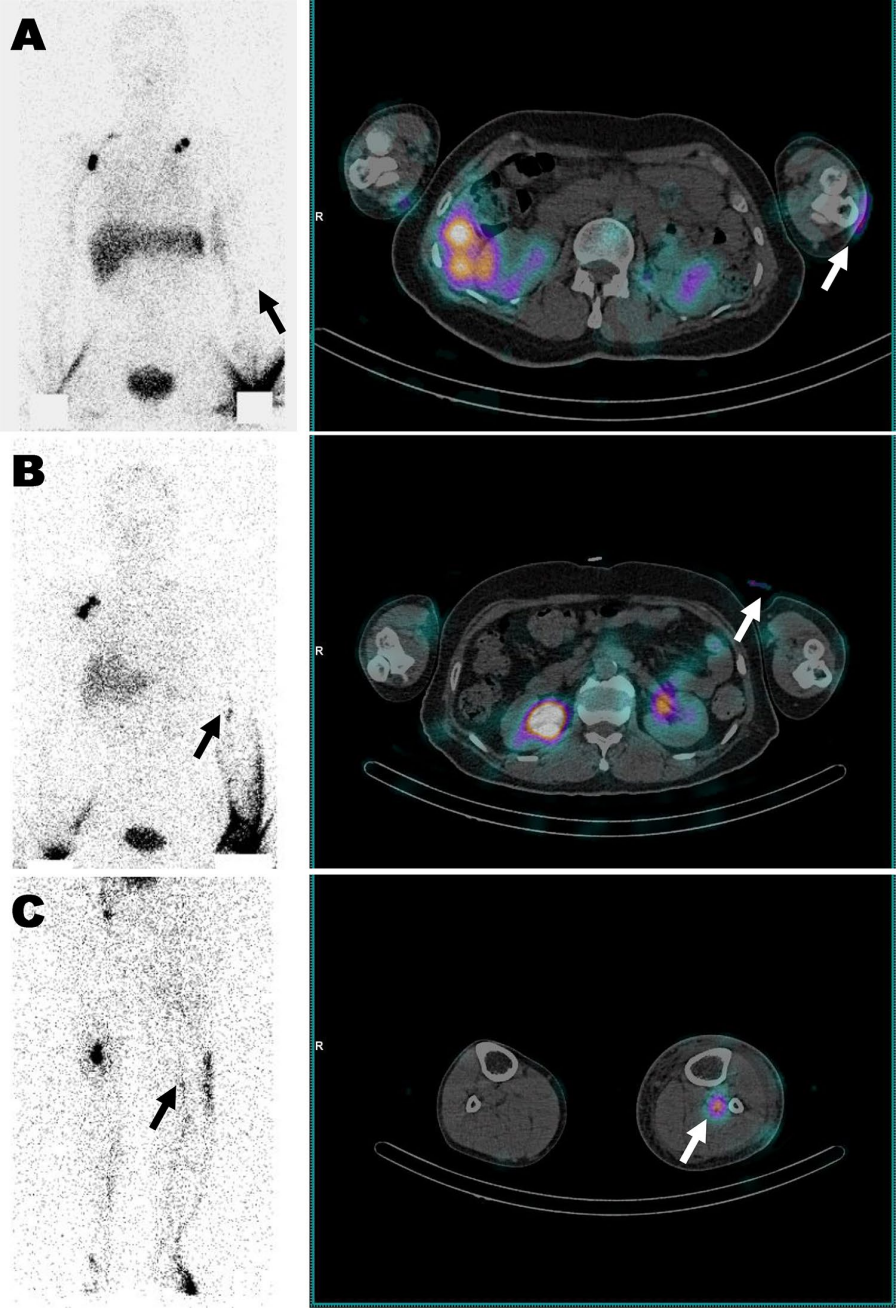
Representative cases of TLS stage change by adding SPECT/CT. Each row presents planar scintigraphy (left) and fused SPECT/CT (right) images of the same patient. The fused SPECT/CT image on the right corresponds to an axial slice at the level indicated in the planar scintigraphy on the left. (**A**) Modification within the stages of partial obstruction: from P-1 (DBF−, black arrow) on planar scintigraphy only to P-2 (DBF+, white arrow) on planar scintigraphy plus SPECT/CT. (**B**) Modification between the stages: from P-3 (intermediate LN+, black arrow) on planar scintigraphy only → T-4 (clothing contamination misinterpreted for intermediate LN, white arrow) on planar scintigraphy plus SPECT/CT. (**C**) Modification between the stages: from T-4 (LN−, black arrow) on planar scintigraphy only to P-3 (intermediate LN+, white arrow) on planar scintigraphy plus SPECT/CT. *TLS* Taiwan lymphoscintigraphy staging, *DBF* dermal backflow.

There were 8 patients with TLS modification between TLS based on planar scintigraphy only and TLS based on planar scintigraphy plus SPECT/CT. Of 8 patients, 2 were modified within the stages of partial obstruction, 2 were modified from the stages of partial to total obstruction, and 4 were modified from the stages of total to partial obstruction. The two patients who were modified within the stages of partial obstruction had DBF detected in SPECT/CT that was not visible on planar scintigraphy. The two patients who were modified from the stages of partial to total obstruction had intermediate lymph nodes suspected on planar scintigraphy, but SPECT/CT showed that the activity was due to contamination from clothing covering the area. The four patients who were modified from the stages of total to partial obstruction had lymph node activity detected in SPECT/CT that was not seen on planar scintigraphy. Excluding the 2 patients where technical artifacts (cloth contamination) led to misleading results, the modification rate of lymphoscintigraphic staging was 15.4% (6/39) (Table 2). However, there was no significant difference in the proportions of the stages between planar scintigraphy only and planar scintigraphy plus SPECT/CT (McNamar’s Chi-squared test, *p* = 0.095). When compared by detailed lymphoscintigraphic finding, the presence of DBF (McNamar’s Chi-squared test, *p* = 0.045) and the visualization of intermediate lymph node (McNamar’s Chi-squared test, *p* = 0.025) showed a significant difference, while the visualization of proximal lymph node (McNamar’s Chi-squared test, *p* = 0.157) and lymphatic duct (McNamar’s Chi-squared test, *p* = 1.000) did not show a significant difference. Representative cases are presented as Fig. 2.

**Table 2:** Contingency table for TLS stage comparison between planar scintigraphy only and planar scintigraphy plus SPECT/CT.

Planar scintigraphy + SPECT/CT		Planar scintigraphy only						
		P-1	P-2	P-3	T-4	T-5	T-6	Total
P-1	N	1	0	0	0	0	0	1
	%	33.33	0.00	0.00	0.00	0.00	0.00	2.56
P-2	N	2	12	0	0	0	1	15
	%	66.67	100.00	0.00	0.00	0.00	12.50	38.46
P-3	N	0	0	4	2	1	0	7
	%	0.00	0.00	100.00	28.57	20.00	0.00	17.95
T-4	N	0	0	0*	5	0	0	5
	%	0.00	0.00	0.00	71.43	0.00	0.00	12.82
T-5	N	0	0	0	0	4	0	4
	%	0.00	0.00	0.00	0.00	80.00	0.00	10.26
T-6	N	0	0	0	0	0	7	7
	%	0.00	0.00	0.00	0.00	0.00	87.50	17.95
Total	N	3	12	4	7	5	8	39
	%	100.00	100.00	100.00	100.00	100.00	100.00	100.00

*Two patients where technical artifacts led to misleading results were excluded.

### Changes in quantitative parameters of respective SPECT and CT according to clinical severity, lymphoscintigraphic staging, and chronicity

**Figure 3:**
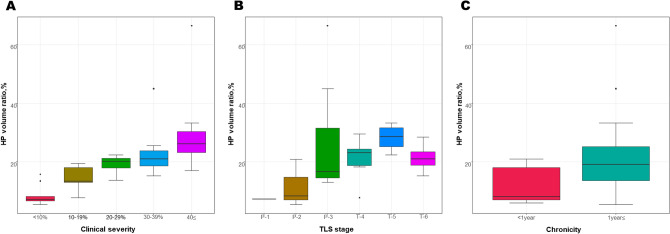
Changes of the HP volume ratio according to (**A**) clinical severity, (**B**) lymphoscintigraphic staging, and (**C**) chronicity. *HP* honeycomb pattern.

The HP volume ratio showed significant differences among clinical severity groups and increased as severity increased (Kruskal–Wallis test, *p* < 0.001) (Fig. 3). The post-hoc pair-wise comparisons revealed differences between Grade 0 and 3 (Bonferroni-adjusted *p* = 0.005), Grade 0 and 4 (Bonferroni-adjusted *p* < 0.001), and Grade 1 and 4 (Bonferroni-adjusted *p* = 0.009). Additionally, the HP volume ratio showed significant differences among lymphoscintigraphic staging groups and increased with stage (Kruskal–Wallis test, *p* = 0.001) (Fig. 3). The post-hoc pair-wise comparisons revealed a difference between P-2 and T-5 (Bonferroni-adjusted *p* = 0.006). Patients with a duration of lymphedema greater than one year were classified into the chronic lymphedema group, and the HP volume ratio was significantly higher in the chronic group (≥ 1 year) than in the non-chronic group (< 1 year) (Mann–Whitney test, *p* = 0.009) (Fig. 3).

**Figure 4:**
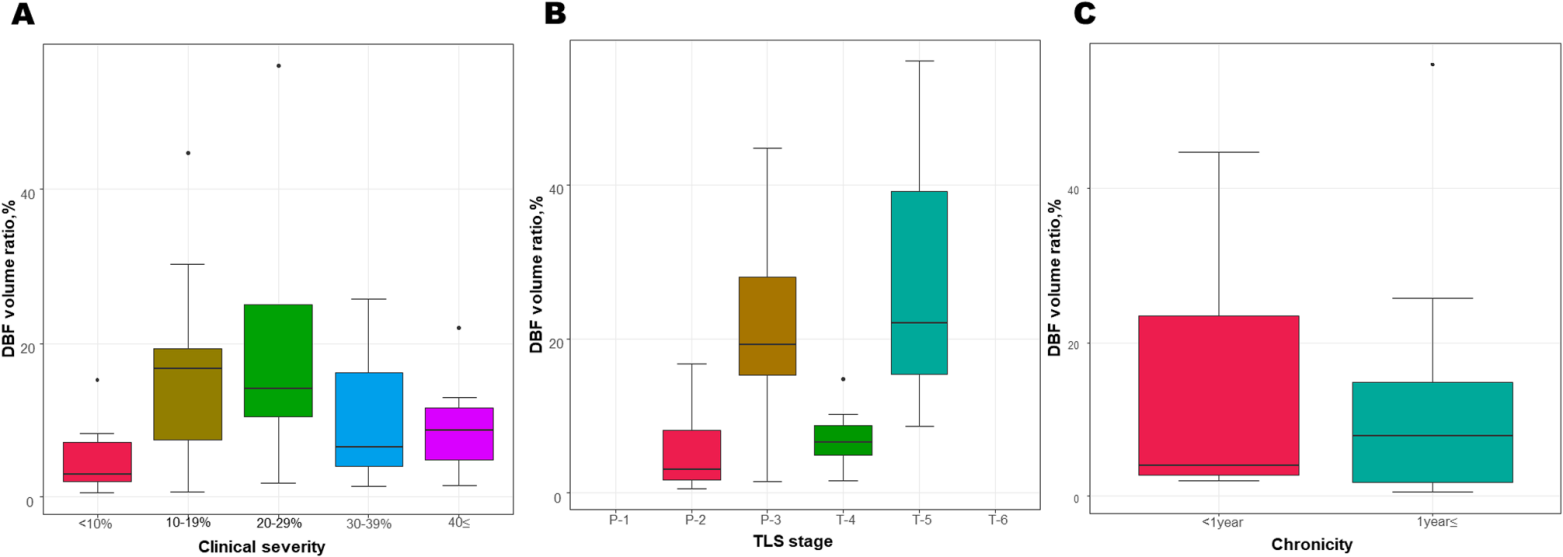
Changes of the DBF volume ratio according to (**A**) clinical severity, (**B**) lymphoscintigraphic staging, and (**C**) chronicity. *DBF* dermal backflow.

The DBF volume ratio did not show a significant difference among clinical severity groups, but it increased with severity and then decreased (ANOVA test, *p* = 0.256) (Fig. 4). However, the DBF volume ratio showed significant differences according to the lymphoscintigraphic staging and followed the expected patterns of increase and decrease according to the staging definition (Kruskal–Wallis test, *p* = 0.008) (Fig. 4). The post-hoc pair-wise comparisons revealed a difference between P-2 and T-5 (Bonferroni-adjusted *p* = 0.029. There was no significant difference according to the chronicity of lymphedema (Mann–Whitney test, *p* = 0.688) (Fig. 4).

### Changes in hybrid SPECT/CT lymphoscintigraphic classification according to clinical severity, lymphoscintigraphic staging, and chronicity

**Figure 5:**
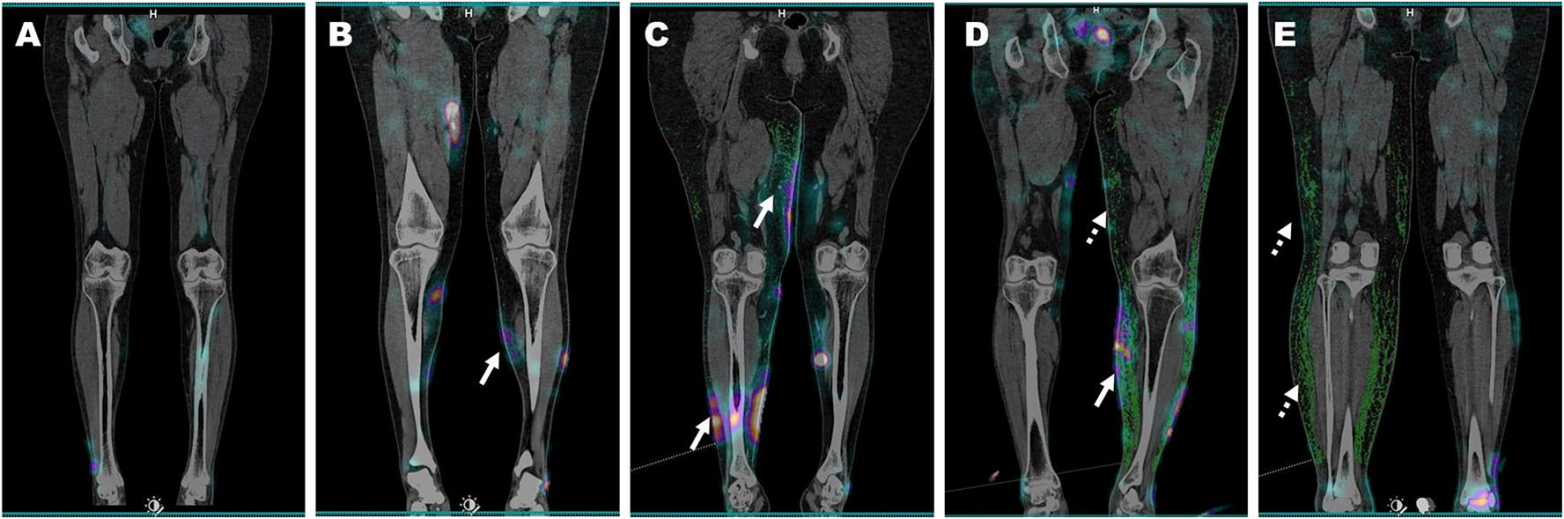
Representative cases according to the hybrid SPECT/CT classification. The coronal view of fused SPECT/CT for each case is presented, with the green area representing segmented HP from the CT image. (**A**) A 70-year-old male patient with lymphedema in the right lower limb. There were no observations of DBF and HP throughout the entire limb, and as a result, it was classified as Class 1 according to the hybrid SPECT/CT classification. The clinical severity was Grade 0, and the TLS stage was P-1. (**B**) A 54-year-old female patient with lymphedema in the left lower limb. DBF was observed in the distal limb, while HP was absent (arrow). As a result, it was classified as Class 2 according to the hybrid SPECT/CT classification. The clinical severity was Grade 0, and the TLS stage was P-2. (**C**) A 68-year-old female patient with lymphedema in the right lower limb. Both proximal and distal limbs showed the presence of DBF accompanied by HP (arrows), and as a result, it was classified as Class 3 according to the hybrid SPECT/CT classification. The clinical severity was Grade 1, and the TLS stage was P-3. (**D**) A 63-year-old female patient with lymphedema in the left lower limb. In the distal limb, DBF accompanied by HP was observed (arrow), while in the proximal limb, HP without DBF was observed (dotted arrow). As a result, it was classified as Class 4 according to the hybrid SPECT/CT classification. The clinical severity was Grade 4, and the TLS stage was T-4. (**E**) A 60-year-old female patient with lymphedema in the right lower limb. Throughout the entire limb, HP was observed without DBF (dotted arrows), and as a result, it was classified as Class 5 according to the hybrid SPECT/CT classification. The clinical severity was Grade 3, and the TLS stage was T-6. *DBF* dermal backflow, *HP* honeycomb pattern, *TLS* Taiwan Lymphoscintigraphy Staging.

**Figure 6:**
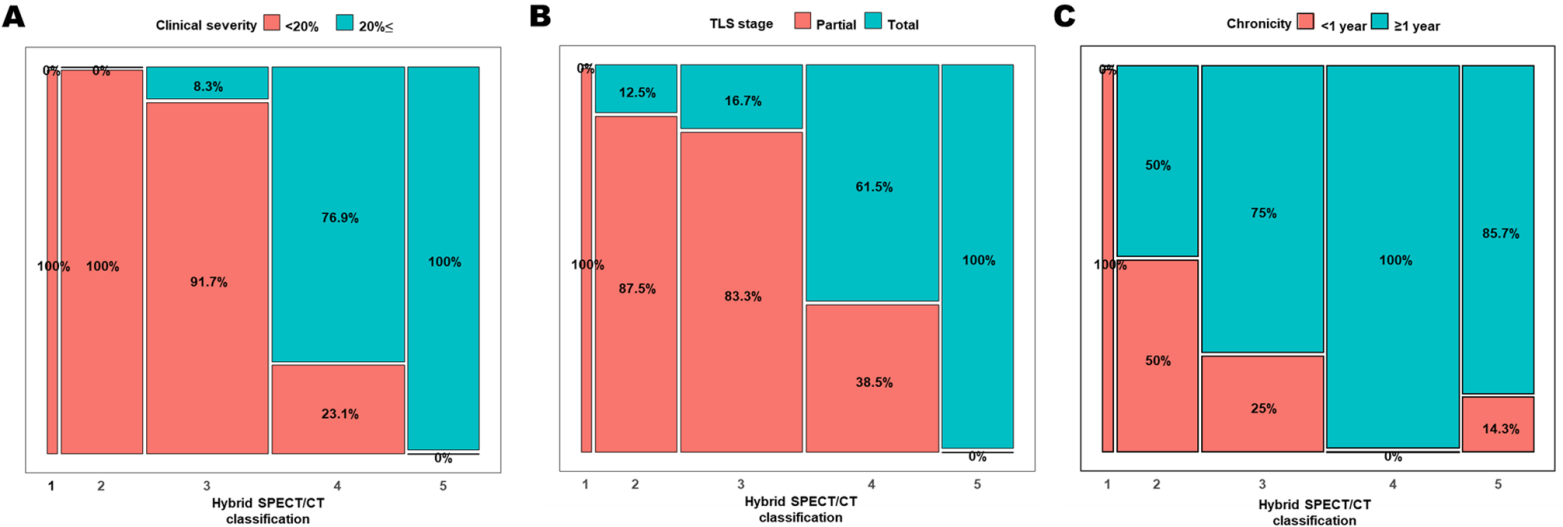
Changes of the hybrid SPECT/CT classification according to (**A**) clinical severity, (**B**) lymphoscintigraphic staging, and (**C**) chronicity.

According to the hybrid SPECT/CT lymphoscintigraphic classification, one patient (2.4%) was classified as Class 1, eight patients (19.5%) as Class 2, twelve patients (29.3) as Class 3, thirteen patients (31.7%) as Class 4, and seven patients (17.1%) as Class 5. Inter-observer agreement for the hybrid SPECT/CT classification was almost perfect (κ = 0.896). Representative cases are presented as Fig. 5. The hybrid classification showed a strong positive correlation with clinical severity (Spearman rank correlation, *r* = 0.814, *p* < 0.001) and TLS staging (Spearman rank correlation, *r* = 0.799, *p* < 0.001) (Supplemental Fig. 1). Additionally, according to the hybrid classification, the proportion of patients in the clinical severity “0–2” group, TLS total obstruction group, and non-chronic group decreased significantly as the classification increased from 1 to 5 (Linear by linear association test, *p* < 0.001 for clinical severity and lymphoscintigraphic staging, *p* = 0.006 for chronicity; Fig. 6).

## Discussion

This study aimed to investigate the value of SPECT/CT lymphoscintigraphy in the initial assessment of secondary lymphedema. We evaluated the lymphoscintigraphic staging changes resulting from the addition of SPECT/CT compared to planar lymphoscintigraphy, based on the TLS stage. We observed considerable modifications in lymphoscintigraphic staging, mostly overcoming the limitations mainly associated with planar imaging, such as finding DBF and LN uptake missed on planar lymphoscintigraphy or skin and clothing contamination misinterpreted for LN intake. These examples demonstrated the advantages of combining SPECT and anatomical CT images to overcome the limitations of planar imaging. Weiss et al. evaluated whether the additional use of SPECT/CT technique improves the diagnostic value of planar lymphoscintigraphy in primary lymphedema^5^. They reported that SPECT/CT combines the superior functional imaging capabilities of SPECT with the anatomical overlay of CT, providing relevant additional information regarding the presence of DBF, the anatomical extent of lymphatic disorders, the presence or absence of LN, and the visualization of lymph vessels. While limited to secondary lymphedema, this study has also identified similar advantages to previous research. Our study acquired delayed images at 1-h post-injection; however, in cases of severely compromised lymphatic transport, there have been instances where additional DBF becomes visible in images obtained beyond 120 min^3,6^. Therefore, delaying the acquisition of delayed images might potentially reduce the impact of lymphoscintigraphic staging modifications resulting from additional SPECT/CT. Further research is needed to investigate this possibility.

Visualizing intermediate or proximal LN is an important factor in determining partial or total obstruction in the TLS staging^3^. Therefore, clearly determining the presence or absence of intermediate or proximal LN can potentially influence treatment decision-making. Additionally, the visualization of intermediate LN, particularly at the elbow or knee, suggests the routing of lymph flow from the superficial to the deep system, providing important information about lymphatic flow^7,8^. It is expected that SPECT/CT will be helpful in providing information about lymphatic flow prior to surgical interventions such as lymphaticovenous anastomosis.

The classic signs of lymphedema observed on CT scan include thickening of the skin, a honeycombed pattern in the subcutaneous tissue, an epifascial fluid lake, and the absence of edema within muscular compartments^9,10^. Among these signs, the honeycombed pattern (HP) in the subcutaneous tissue is believed to be caused by the presence of fibrotic tissue and fluid surrounding the accumulation of adipose tissue. HP is often considered specific to lymphedema as it is not found in conditions like lipodystrophy or edema of venous origin^9^. Vaughan et al. have also proposed an association between HP and chronic inflammation, characterized by an influx of macrophages and collagen deposition by fibroblasts^11^. In this study, HP volume ratio showed significant differences based on clinical severity, lymphoscintigraphic staging, and chronicity. As clinical severity increased, imaging staging became higher, and the duration of the condition was longer, the HP volume ratio significantly increased. This finding aligns with the results of Yoo et al., which reported a higher prevalence of HP in more severely affected lymphedema and in chronic cases^10^. It should be noted that these fluid densities can be reversed, as previously reported after complex physiotherapy, indicating that the presence of a HP alone is not indicative of lymphatic drainage efficacy or lack of response to treatment^9^. Reports stating that HP is not predictive of lymphatic drainage efficacy and does not signify lack of response to treatment suggest that HP alone is insufficient to explain the progression of lymphedema.

DBF, observed as a diagnostic finding of lymphedema on lymphoscintigraphy, is characterized by dilated dermal lymphatic capillaries and pre-collectors^12^. It develops when the obstruction of superficial lymph-collecting vessels leads to incompetence of the valves in the pre-collectors. DBF is considered the body's anatomical response to lymphatic obstruction, creating alternative pathways for lymph fluid transport^13,14^. Some studies have reported that the amount of DBF in the limbs correlates with the severity of lymphedema^15,16^. However, unlike HP, DBF may be minimal or absent in the final stages, indicating non-functional lymphatics^3,14^. Additionally, DBF can be present from the subclinical or latent phase. Akita's prospective cohort study of ICG lymphography in breast cancer patients confirmed that DBF could be identified even in patients in the latent phase of lymphedema, where no clinical symptoms were evident^17^. In this study, the measured DBF volume ratio did not show statistically significant differences between groups based on clinical severity and chronicity. However, it demonstrated a trend of increasing with severity and then decreasing towards the end. This finding aligns with the staging criteria of indocyanine green (ICG) fluorescence lymphography and lymphoscintigraphy, which include DBF as an indicator^3,14^. We scored the lymphoscintigraphic findings according to the TLS staging, which is one of the various known lymphoscintigraphic staging methods. We observed significant differences between groups based on the staging criteria. Post hoc tests revealed that the significance of the differences between stages was driven by the comparison between P-2 and P-3. This finding reflects the definition of TLS stages, where P-2 represents proximal or distal DBF and P-3 represents entire DBF. While DBF is considered a negative pathological change in lymphedema due to patient discomfort and skin induration, it is a positive change in anatomical terms as it helps maintain lymph drainage by creating an alternative pathway. In other words, the presence of DBF indicates that the lymphatic system is functioning, albeit in a modified state.

Baulieu et al. analyzed the correlation between DBF and HP and reported no significant correlation between the two parameters^18^. Consistent with these findings, in our analysis, DBF and HP did not show any association (Supplemental Fig. 2, *p* = 0.321 and *r* = 0.180). This is an expected result considering that DBF and HP exhibit different patterns based on clinical severity, TLS staging, and duration of the condition. DBF appears early, increases with severity or staging, and eventually disappears, while HP increases proportionally in the later stages. Based on the existing literature and the quantitative analysis results of our study, we developed an SPECT/CT classification combining DBF and HP and analyzed its changes based on clinical severity, TLS staging, and chronicity. The hybrid SPECT/CT classification showed a strong positive correlation with clinical severity and lymphoscintigraphic staging. Pecking et al. conducted a study to determine whether the results of conventional lymphoscintigraphy and fusion imaging obtained from hybrid detectors could be used for a comprehensive clinico-imaging staging in extremity lymphedema^6^. They found that CT imaging, when combined with clinical assessment and lymphoscintigraphy, was useful in evaluating tissue changes associated with lymphedema in patients with lower limb lymphedema. The combination of clinical, lymphoscintigraphic, and CT staging was found to have predictive value for treatment efficacy. The classification proposed in this study differs from Pecking's study in that it incorporates the course of lymphatics (superficial vs. deep) into the staging process and utilizes SPECT/CT imaging to differentiate between deep and superficial lymphatics. However, it should be noted that evaluating dynamic lymphatic flow through SPECT/CT at a single time point has its limitations. CT imaging, in this context, primarily serves as a supplementary tool to indicate the anatomical location (superficial vs. deep) of lymphatic structures. On the other hand, this study introduces a new comprehensive evaluation criterion by combining SPECT and CT indicators, which represent distinct pathophysiological aspects and have no direct correlation with each other. By incorporating both imaging modalities, the proposed criterion aims to provide a more comprehensive assessment of lymphedema.

In summary, SPECT/CT lymphoscintigraphy overcomes the limitations of planar imaging, allowing for more accurate staging. Furthermore, it provides a more accurate assessment by considering factors such as the extent of dermal backflow, progression of fibrosis, as well as lymph node function, in determining surgical indications for lymphedema. Additionally, it is expected to be valuable in evaluating treatment responses through precise pre- and post-treatment assessments.

However, lymphoscintigraphic staging is part of evaluating the clinical progression of lymphedema and does not always result in changes to treatment plans, including surgery. Treatment decisions are influenced by a comprehensive assessment that takes into consideration clinical severity, ICG findings, and other factors, and there is currently no consensus on indications. Prospective studies to assess whether changes in lymphoscintigraphic staging due to the addition of SPECT/CT ultimately impact treatment decisions are promising. Additionally, consideration should be given to the increased radiation dose and cost–benefit analysis associated with the addition of CT.

This study has several limitations. First, the sample size was small. Despite the limited number of patients, the addition of SPECT/CT overcame the limitations of planar lymphoscintigraphy and demonstrated considerable modifications in lymphoscintigraphic staging, which is meaningful. Further observational studies or meta-analyses with larger sample sizes should be considered. Second, the distribution of patients across ISL stages is uneven. The relatively small patient numbers in ISL Stage 1 and 3 could potentially impede the observation and identification of the HP in advanced lymphedemas and DBF in patients with very early-stage lymphedemas. Third, based on the qualitative and quantitative analysis results of this study alone, it is not possible to determine whether DBF or HP occurs first in the progression of lymphedema. Conducting preclinical studies using animal models with periodic imaging examinations would be beneficial in addressing this question. Finally, this study focuses on the analysis of secondary lymphedema. While the utility of SPECT/CT has been previously reported in primary lymphedema^5^, further research is needed to determine whether the application of the hybrid SPECT/CT classification will yield similar results in primary lymphedema.

## Methods

### Patients

We retrospectively evaluated patients with extremity lymphedema who underwent SPECT/CT lymphoscintigraphy from April 2022 to December 2022, at Ewha Womans University Medical Center in Seoul, South Korea. This study included patients who underwent SPECT/CT lymphoscintigraphy as part of the treatment planning for lymphedema. We excluded patients who underwent imaging for follow-up after reconstructive surgery of the lymphatic system, those with primary lymphedema, bilateral lymphedema, and cases with incomplete imaging. All clinical data, such as age, sex, body mass index, duration of lymphedema, site of lymphedema, and previous cancer or cancer-related treatment history were collected by reviewing the electronic medical records. The clinical severity of extremity lymphedema was assessed using CT volumetry, according to the method described below. This study was conducted in accordance with the Declaration of Helsinki and approved by the institutional review board of Ewha Womans University Mokdong Hospital with a waiver of informed consent (IRB no. 2023-03-020).

### Imaging protocol of SPECT/CT lymphoscintigraphy

Planar and SPECT/CT images were acquired using a SPECT/CT hybrid system (Symbia Intevo Bold, Siemens Healthineers) equipped with low-energy high-resolution collimators. The matrix size was 512 × 512 and 256 × 1024 for the regional static and whole-body images, respectively. The acquisition speed was 15 cm/min and zoom factor was 1.0. The patient was placed in the supine position, and 37 MBq (1 mCi) of Tc-99m phytate in a volume of 0.4 mL was injected intradermally using a 27-gauge syringe in the second and third webspace of both hands or the first and second webspace of both feet. Early lymphatic flow images were obtained at 5 min after injection and followed by delayed images at 30 min and 1-h post-injection in both extremities. In addition to planar imaging, SPECT/CT images were acquired over 20 min with the following parameters: 256 × 256 matrix, rotation of 180°, 6° view angle, 20 s per projection. In our institution, SPECT/CT scans has been routinely performed in all patients undergoing lymphoscintigraphy. The patients were asked to walk or clench and unclench their hands for 20 min between the image acquisitions to improve the transport of the radiopharmaceutical.

### SPECT/CT lymphoscintigraphy assessment with Taiwan Lymphoscintigraphy Staging system

According to a previously published study^19^, lymphoscintigraphic findings were evaluated using the Taiwan Lymphoscintigraphy Staging (TLS), which were applied to both upper and lower extremities. Two board-certified nuclear medicine physicians (H.-J.Y. and J.Y.K.) who were blinded to any clinical information graded the lymphoscintigraphic stage according to TLS independently, and a consensus was reached. Each reader reviewed two image sets, planar scintigraphy alone and planar scintigraphy plus SPECT/CT, with a one-month interval between each review. Lymphoscintigraphy was classified into three patterns based on the visualization of proximal/intermediate lymph nodes, linear lymphatic ducts, and the extent of dermal backflow (DBF), which were further subdivided into seven stages: normal drainage (L-0), partial obstruction (P-1, P-2, and P-3), and total obstruction (T-4, T-5, and T-6). We compared the TLS stages evaluated with the set consisting of planar scintigraphy alone and the set consisting of planar scintigraphy plus SPECT/CT to investigate whether there was any difference between the two image sets.

### CT-based quantitative analysis

The volume of the extremity was measured from combined CT using the segmentation tool called region growing provided by syngo.via VB60A (Siemens Healthineers). The boundaries for the volumetric segmentation of the lower limbs were set from the perineum to the ankle, while the upper limbs were set from the axillary crease to the wrist^20^. The total volume (cm^3^) was automatically extracted from the segmented extremity using automatic contouring and interpolation functions. The CT volumetric difference was calculated by dividing the difference between the total volume of the affected extremity and the total volume of the unaffected extremity by the total volume of the unaffected extremity, with five grades defined as follows: Grade 0 (< 10%), Grade 1 (10%–19%), Grade 2 (20%–29%), Grade 3 (30%–39%), and Grade 4 (40% ≤)^19^. To segment the honeycomb pattern (HP) in the subcutaneous (SC) tissue, we applied a threshold range of − 60 to 10 Hounsfield unit (HU) on the segmented SC^10^. The segmentation of the SC tissue was obtained by simply subtracting segmented muscle and bone from the segmented extremity (Supplemental Fig. 3). The ratio of HP volume to SC volume of affected extremity was defined as the HP volume ratio and was used in the subsequent analysis.

### SPECT-based quantitative analysis

We used the PMOD 4.2 (PMOD Technologies, LLC) to extract the volume of dermal backflow (DBF) from 3-dimensional SPECT. The volume of interest (VOI) for DBF was defined using the 3D iso-contouring option based on region growing. The resulting volume was automatically calculated and used to determine the ratio of DBF volume to SC volume of the affected extremity, which was defined as the DBF volume ratio for subsequent analysis.

### Hybrid SPECT/CT lymphoscintigraphic classification

The SPECT/CT findings were classified into four patterns: DBF−/HP− = the absence of both DBF and HP in the SC compartment, DBF+/HP− = the presence of DBF and the absence of HP in the SC compartment, DBF+/HP+ = the presence of both DBF and HP in the SC compartment, and DBF−/HP+ = the absence of DBF and the presence of HP in the SC compartment (Supplemental Fig. 4). Based on these findings, patients were categorized into five classes: Class 1 = No observation of DBF and HP (DBF−/HP−) in the entire affected extremity, Class 2 = Presence of DBF+/HP− in the proximal or distal extremity, with no presence of DBF+/HP+ or DBF−/HP+ in the entire affected extremity, Class 3 = Presence of DBF+/HP+ in the proximal or distal affected extremity, with no presence of DBF−/HP+ in the entire affected extremity, Class 4 = Coexistence of DBF+/HP+ and DBF−/HP+ in the proximal or distal affected extremity, Class 5 = Presence of HP+ without DBF (DBF−/HP+) in the entire affected extremity.

Inter-observer agreement for the hybrid SPECT/CT classification was evaluated using weighted κ statistics. The κ value was interpreted as follows: 0.00–0.20 poor agreement, 0.21–0.40 fair agreement, 0.41–0.60 moderate agreement, 0.61–0.80 substantial agreement, and 0.81–1.00 almost perfect agreement.

### Statistical analysis

Descriptive statistical analyses were performed for all patients included in the study. Normality test was performed by Shapiro–Wilk test. Numerical data were expressed as mean with standard deviation (SD) if the normality test was accepted, and as median with interquartile range (IQR) if the normality test was rejected. To evaluate whether there is a significant difference in the proportions of lymphoscintigraphic staging between planar scintigraphy alone and planar scintigraphy plus SPECT/CT, McNamar’s Chi-squared test was used. Based on the results of the normality test, Student *t*-test or Mann–Whitney test was used for the comparison of continuous variables between two groups. For the comparison among more than three groups, ANOVA test or Kruskal–Wallis test was used and followed by post-hoc pair-wise comparisons. For the comparison of categorical variables, Chi-squared test or Fisher’s exact test was used. Spearman rank correlation and linear by linear association test were used to analyze the correlation between hybrid classification and clinical severity as well as TLS staging. All statistical tests were 2-sided with a significance threshold of *P* < 0.05. Data analyses were performed using two commercial software programs (version 26.0, IBM SPSS Statistics, Armonk, NY, USA; Rex 3.6.0, Rexsoft, Seoul, Korea).

### Supplementary Information

Supplementary Figures.

## Data Availability

The datasets used and/or analyzed during the current study are available from the corresponding author on reasonable request.
