# Supplementary material for: The added value of SPECT/CT lymphoscintigraphy in the initial assessment of secondary extremity lymphedema patients
Source: Sci Rep. 2023 Nov 9;13:19494. doi: 10.1038/s41598-023-44471-2 (PMC10636025; doi:10.1038/s41598-023-44471-2)
Supplement: Supplementary file 1 — Supplementary Figures. [file 41598_2023_44471_MOESM1_ESM.docx]

**Supplemental Figure 1.** **Changes in hybrid SPECT/CT lymphoscintigraphic classification according to lymphoscintigraphic staging (A) clinical severity (B)**


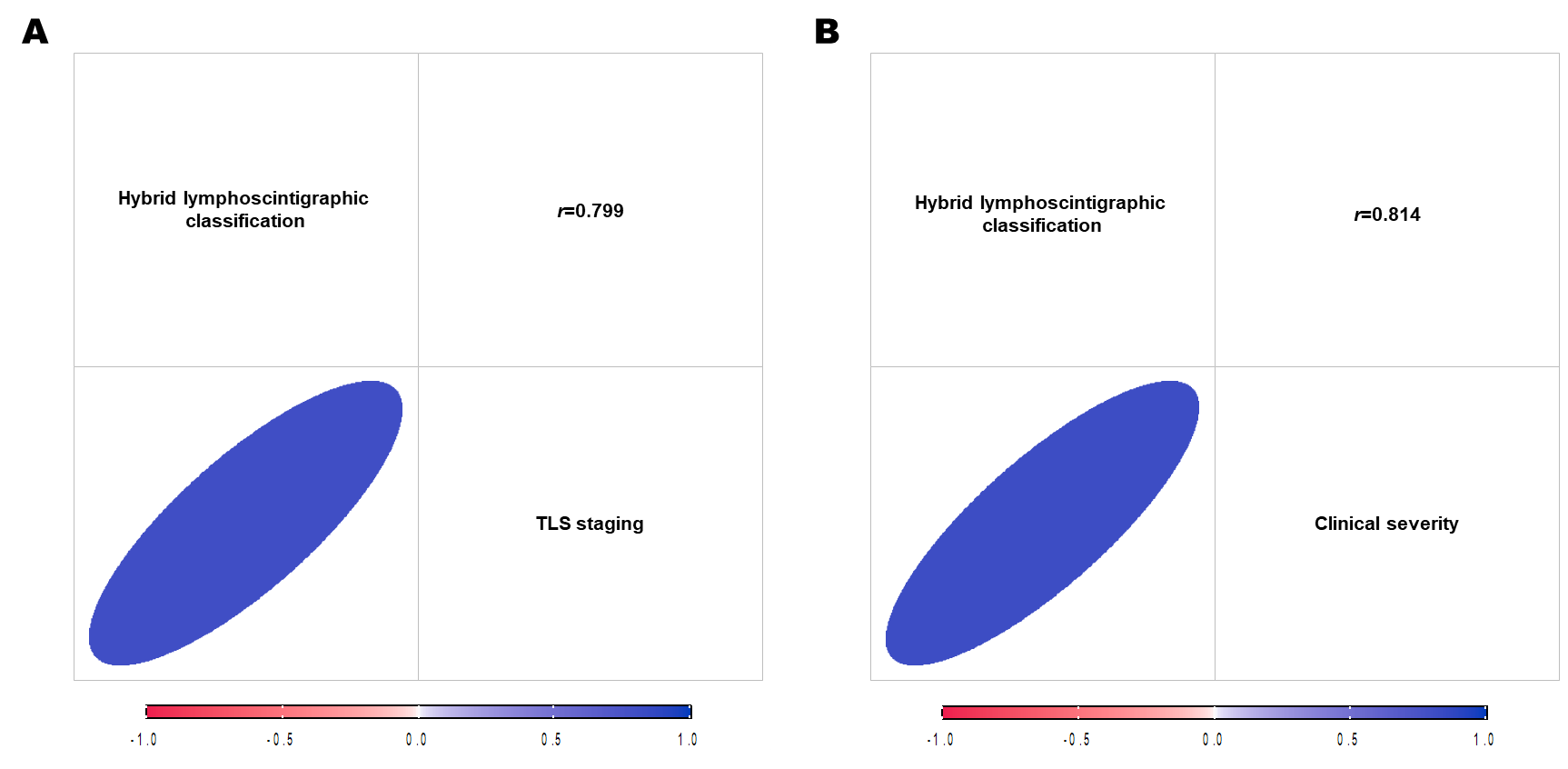


**Supplemental Figure 2. Correlation between DBF volume ratio and HP** **volume ratio**


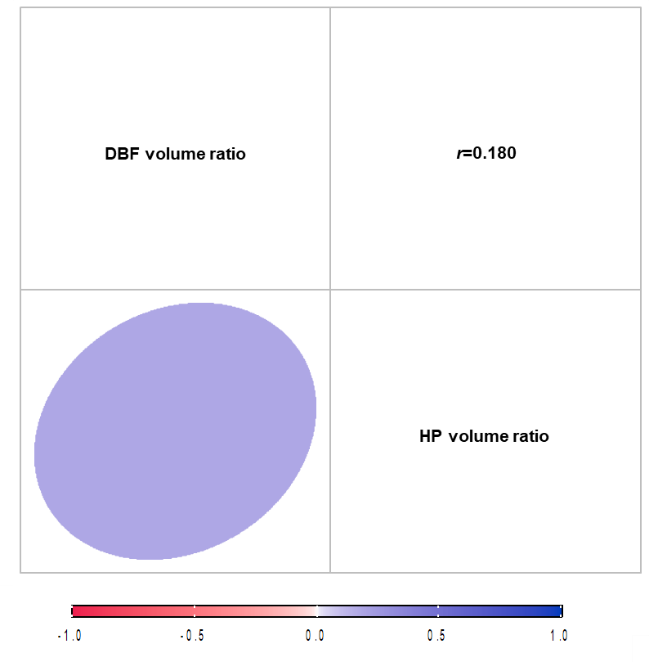


**Supplemental Figure 3. Algorithmic CT-based quantitative volume measurement.**


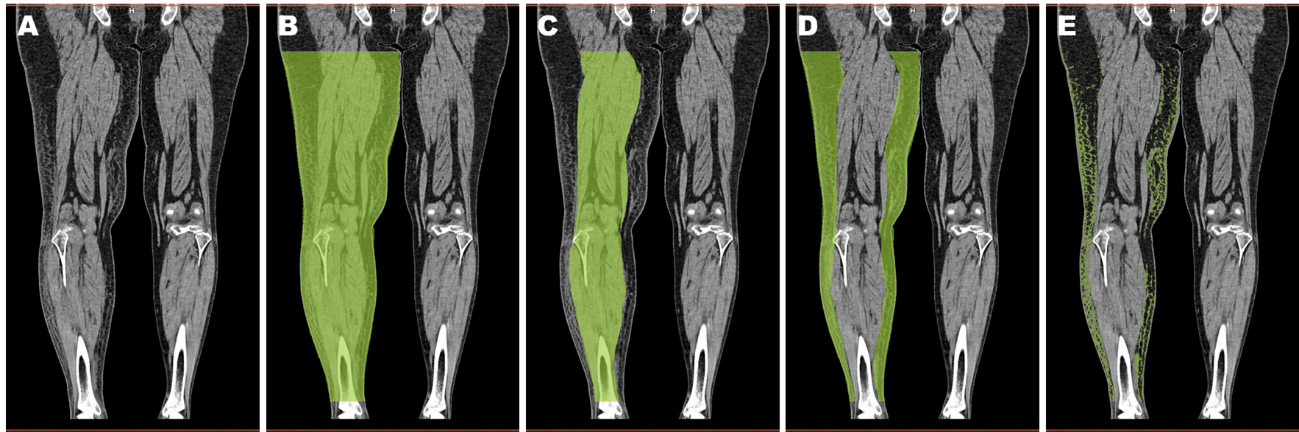


(A) CT dicom images were revrieved. (B) Total volume from the perineum to the ankle was segmented using automatic contouring and interpolation functions. (C) Muscle and bone volume were segmented from the total volume using automatic contouring and interpolation functions. (D) SC volume was obtained by subtracting the segmented muscle and bone volume from the total volume (B-C). (E) A threshold range of -60 to 10 HU was applied to the segmented SC (D) to identify and segment the honeycomb pattern within the SC compartment.

**Supplemental Figure 4. Illustrations showing the pattern of SPECT/CT.**


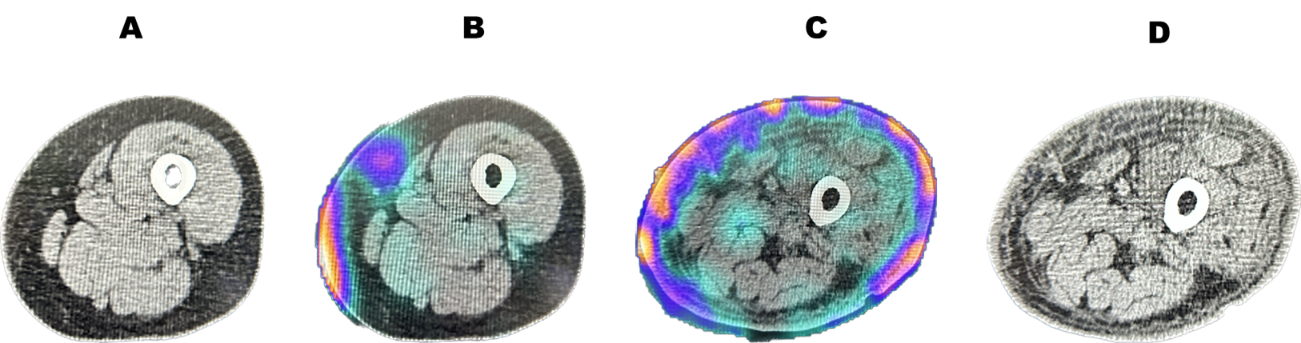


(A) DBF-/HP-: the absence of DBF and the absence of HP in the SC compartment. (B) DBF+/HP-: the presence of DBF and the absence of HP in the SC compartment. (C) DBF+/HP+: the presence of both DBF and HP in the SC compartment. (D) DBF-/HP+: the absence of DBF and the presence of HP in the SC compartment.
